# Robotic pancreaticoduodenectomy after endoscopic ultrasound-guided choledochoduodenostomy for malignant biliary obstruction

**DOI:** 10.1055/a-2975-6279

**Published:** 2026-10-26

**Authors:** Marco Spadaccini, Giovanni Capretti, Miriana Mercurio, Greta Donisi, Matteo Colombo, Alessandro Zerbi, Alessandro Repici

**Affiliations:** 1 Endoscopy Unit, Gastroenterology Department 9268 IRCCS Humanitas Research HospitalRozzano, MilanItaly; 2 Department of Biomedical Sciences 437807 Humanitas UniversityPieve Emanuele, MilanItaly; 3 Pancreatic Surgery Unit 9268 IRCCS Humanitas Research HospitalRozzano, MilanItaly

Endoscopic ultrasound-guided choledochoduodenostomy (EUS-CDS) is an effective treatment option for malignant distal biliary obstruction. 1​ Its role has further expanded with the introduction of cautery-enhanced lumen-apposing metal stents (LAMSs), which have demonstrated higher technical success rates and shorter procedure times than self-expandable metal stents (SEMSs), with comparable clinical success and overall adverse event rates, supporting their use for efficient biliary drainage in this setting. 2​ EUS-CDS has also emerged as a viable primary drainage strategy and as an early alternative in patients with difficult biliary cannulation. 3​ ​4​ However, evidence regarding the impact of preoperative EUS-CDS on subsequent pancreaticoduodenectomy, particularly when performed using a minimally invasive approach, remains limited. 5​

We report the case of a patient with histologically confirmed pancreatic head adenocarcinoma who presented with acute cholangitis secondary to malignant distal biliary obstruction. Primary EUS-CDS was performed with direct deployment of an 8×8 mm cautery-enhanced LAMS. The procedure was technically successful, with no procedure-related adverse events, and resulted in clinical improvement and resolution of cholangitis.

Follow-up abdominal computed tomography demonstrated biliary decompression and confirmed tumor resectability. The patient subsequently underwent robotic pancreaticoduodenectomy. The LAMS was removed intraoperatively without technical difficulties or complications ( Video 1 ), and the postoperative course was uneventful.

**Video 1** Endoscopic ultrasound–guided choledochoduodenostomy resolved cholangitis from resectable pancreatic cancer, enabling robotic pancreaticoduodenectomy with uncomplicated intraoperative lumen-apposing metal stent removal. URI:


This case illustrates that, in selected patients with resectable pancreatic head adenocarcinoma and malignant distal biliary obstruction, EUS-CDS with a cautery-enhanced LAMS can provide effective preoperative biliary drainage without precluding subsequent curative-intent robotic pancreaticoduodenectomy.

Endoscopy_UCTN_Code_TTT_1AS_2AH

## Declaration of GenAI Use

The authors confirm that no generative artificial intelligence was used in the preparation of this manuscript.
